# 9.J. Round table: Experiences on infodemic management in public health authorities in Europe and internationally

**DOI:** 10.1093/eurpub/ckac129.585

**Published:** 2022-10-25

**Authors:** Organised by: PHIRI, EUPHA-PHMR, EUPHA-LAW, WHO, Chair persons: Petronille Bogaert (EUPHA-PHMR), Tim Nguyen (WHO Europe)

## Abstract

The COVID-19 pandemic and current recovery efforts have been complicated by a parallel infodemic, an overwhelming amount of information, including mis- and disinformation, accompanying emergencies as individuals and communities struggle to separate scientific facts and guidance from manipulative, emotionally charged or inaccurate content. The infodemic has manifested itself in the rapid spread of questions, concerns and misinformation that can affect population attitudes and behavior harmful to health-from promoting stigma and discrediting science, to promoting alternative, non-recommended treatment and cures to politicizing public health programs and eroding trust in healthcare personnel and health system. Since the beginning of the COVID-19 pandemic, major advances in the nascent field of infodemiology and the practice of infodemic management have been made, with over 80% of WHO Member States reporting in a pulse survey that they are tracking COVID-19 misinformation and doing infodemic management work. Infodemic management, analogous to epidemic management, is an evidence-based practice in detecting, characterising, responding and managing the infodemic and its harmful effects. It is a leading area of concern for Ministries of Health, who have established infodemic management teams and insights units to help inform programmatic and communications shifts in face of a constantly evolving infodemic. During the COVID-19 crisis and ensuing recovery efforts in face of competing health priorities, public health authorities have been challenged in the way they engage with the public. Advances in social media and media consumption that have eased information sharing between people and communities have also become areas for infodemic risk, such as closed messaging apps, minimally regulated social media platforms, a noisy media environment and under-resourced communications and community engagement functions of MoH and IPH staff who are not versed in technology. The Population Health Information Research Infrastructure (PHIRI) generates and supports their partners in the generation of evidence for research on health and well-being of populations impacted by COVID-19. It supports exchange of expertise across Europe in the area of infodemic management as well.

This workshop aims to share the experiences with infodemic management during the pandemic and key learnings to take forward during recovery and future health systems strengthening and pandemic preparedness efforts. It will be organised as a round table, where speakers will present on how their health systems have built infodemic management capacity, what lessons they have learned and plan to apply to future efforts. Common themes from the participants will become a frame for the Q&A and for audience members to submit questions and their own lessons learned for discussion on these themes through an interactive interface.

**Key messages:**

• Infodemic management is growing from nascent science into full-fledged and more integrated public health practice ripe for innovation and application to health topics beyond COVID-19.

• Implementation science and evaluation of what works and doesn’t in infodemic management must be systematically used for preparedness and response and improvement of routine health service delivery.

**Speakers/Panellists:**

**Elena Petelos**

CSFM & HSR-PH Lab, Faculty of Medicine, University of Crete, Iraklion, Greece

**Christina Leuker**

Robert Koch Institute, Berlin, Germany

**Neville Calleja**

Department of Health Information and Research, University of Malta, Msida, Malta

**Cherstyn Hurley**

UK Health Security Agency, London, UK

**Stefan Mandić-Rajčević**

University of Belgrade, Faculty of Medicine, Belgrade, Serbia

